# Supplementary material for: The Systems Biology Research Tool: evolvable open-source software
Source: BMC Syst Biol. 2008 Jun 29;2:55. doi: 10.1186/1752-0509-2-55 (PMC2446383; doi:10.1186/1752-0509-2-55)
Supplement: Additional file 1 — SBRT Archive. An archive of the current version of the Systems Biology Research Tool. [file 1752-0509-2-55-S1.zip › sbrt-1.4.0/doc/developers_guide/api/sbrt/shell/io/XmlFileFactory.html]

XmlFileFactory


|  |  |  |
| --- | --- | --- |
| **Overview** \| **Package** \| **Class** \| **Use** \| **Tree** \| **Deprecated** \| **Index** \| **Help** | |  |
| **PREV CLASS**   NEXT CLASS | **FRAMES**    **NO FRAMES**     **All Classes** |  |
| SUMMARY: NESTED \| FIELD \| CONSTR \| METHOD | DETAIL: FIELD \| CONSTR \| METHOD |  |


---


## sbrt.shell.io Class XmlFileFactory

```
java.lang.Object
  sbrt.shell.io.XmlFileFactory
```

---

``` public class XmlFileFactory extends java.lang.Object ```

This class is used to create DOM objects from XML files.

**Author:**
:   This class was written and documented by Jeremiah Wright while in the
    Wagner lab.

---

**Constructor Summary**

`XmlFileFactory()`


| **Method Summary** | |
| --- | --- |
| `static org.w3c.dom.Document` | `getDOM(java.lang.String fileName)`             Returns a DOM object for the file with the specified name. |

**Methods inherited from class java.lang.Object**

`clone, equals, finalize, getClass, hashCode, notify, notifyAll, toString, wait, wait, wait`

**Constructor Detail**

### XmlFileFactory

```
public XmlFileFactory()
```


**Method Detail**

### getDOM

```
public static org.w3c.dom.Document getDOM(java.lang.String fileName)
```

:   Returns a DOM object for the file with the specified name.

    :   **Parameters:**: `fileName` - the name of the XML file. **Returns:**: a DOM object for the file with the specified name. **Throws:**: `ApplicationException` - if any error occurs while reading or parsing the specified file.


---


|  |  |  |
| --- | --- | --- |
| **Overview** \| **Package** \| **Class** \| **Use** \| **Tree** \| **Deprecated** \| **Index** \| **Help** | |  |
| **PREV CLASS**   NEXT CLASS | **FRAMES**    **NO FRAMES**     **All Classes** |  |
| SUMMARY: NESTED \| FIELD \| CONSTR \| METHOD | DETAIL: FIELD \| CONSTR \| METHOD |  |


---
